# Surviving the storm: A 6-year journey with bowel perforations and aneurysms in vascular Ehlers-Danlos syndrome — A case report

**DOI:** 10.1016/j.ijscr.2024.110294

**Published:** 2024-09-14

**Authors:** Farid Abi Habib, Michael Osseis, Alain Chebly, Elie El Feghali, Roger Noun

**Affiliations:** a Department of Digestive Surgery, Hotel Dieu de France Hospital, Saint Joseph University – Faculty of Medicine, Beirut, Lebanon; b Center Jacques Loiselet for Medical Genetics and Genomics (CGGM), Faculty of Medicine, Saint Joseph University, Beirut, Lebanon

**Keywords:** Ehlers Danlos, Aneurysms, Bowel perforation, COL3A1

## Abstract

**Introduction:**

Throughout the literature, many cases of vascular Ehlers-Danlos were reported with a variation of its clinical presentation. This disease may present in many forms and aspects and some even die before seeking medical care and getting the right diagnosis due to its hard effect on patients.

**Case presentation:**

We report a case of a 25-year-old female patient with a history of multiple bowel perforations that were operated on urgently numerous times and received many courses of a large spectrum of antibiotics for various infections.

**Clinical discussion:**

Further investigations showed that the patient had multiple aneurysms due to her disease, a hepatic aneurysm, a paraspinal, and a severally symptomatic wrist aneurysm. The patient presented a de novo variant in the COL3A1 gene. A management plan was personalized, and a family genetic investigation was carried out.

**Conclusion:**

This article contains a full history of this patient including all the surgical, medical, and radiological interventions.

## Highlights

• The coexistence of multiple organ involvement in a vascular Ehlers Danlos requires a multidisciplinary approach.
• Early diagnosis and intervention can improve the patient's quality of life.
• Genetic counseling for early detection in the patient's family can enhance management and interventions.

## 1 Introduction

Vascular Ehlers-Danlos syndrome (vEDS) is characterized by arterial, intestinal, and uterine fragility; thin, translucent skin; easy bruising; characteristic facial appearance; and an aged appearance to the extremities, particularly the hands. Vascular dissection or rupture, gastrointestinal perforation, or organ rupture are the presenting signs in most adults with vEDS [1]. Also known as congenital connective tissue hypoplasia syndrome, Ehlers-Danlos Syndrome (EDS) represents a class of collagen disorders among the wider group of heritable connective tissue diseases [2].

## 2 Methods

The article discusses a 25-year-old patient who was diagnosed with vascular Ehlers-Danlos syndrome (vEDS), a rare genetic disorder, in a tertiary care hospital located in Beirut, Lebanon. The patient's case was notable for its atypical presentation, making it a challenging diagnosis. Consent for the case study was obtained from both the patient and her family, ensuring that all ethical considerations were met. The report has been meticulously documented following the SCARE criteria, which provide guidelines for the structured reporting of surgical and clinical case studies. [3]

## 3 Case presentation

This is the case of a 25-year-old female patient with a history of undocumented hemoptysis in early childhood and repair of varicose veins in the lower extremities who presented in December 2018 with acute abdominal pain. The patient was diagnosed with acute peritonitis, and an urgent laparotomy revealed a perforated sigmoid colon, leading to an urgent Hartmann procedure. She was discharged six weeks later with a functional colostomy after receiving a course of antibiotics.

Seven months later, the patient was admitted for closure of the colostomy. An end-to-end anastomosis was performed without complications. The basic workup showed no abnormalities in her labs, and investigations at that time (no genetic test was done) came back negative.

In August 2023, the patient presented with acute abdominal pain. An urgent CT scan revealed peritonitis, leading to urgent admission to the ICU. A subsequent urgent laparotomy showed a perforation in the descending colon, next to the previous anastomosis. An extended colectomy was performed, and a transverse colostomy was created. Multiple small bowel perforations were also present and treated with resection-anastomosis. The patient lost about 1.5 l of blood during the operation, for which she received multiple transfusions. She was admitted to the ICU for two weeks, with a total hospital stay of two months. This stay was complicated by various infections and wound infections, as well as enterocutaneous fistulas, which were managed conservatively.

Pathology results showed multiple perforations in the resected small bowel and colonic wall, with marked inflammatory infiltrate from the mucosa to the serosal surface, covered by fibrinopurulent exudate containing clusters of giant cells. The bowel wall showed focal thinning of the muscular propria and dilated congested submucosal and serosal blood vessels. Because of these unusual findings in pathology results, the patient was then tested for genetic disease diagnosed with vEDS, and was prescribed a multivitamin regimen along with a daily dose of vitamin C.

**Fig. 1:**
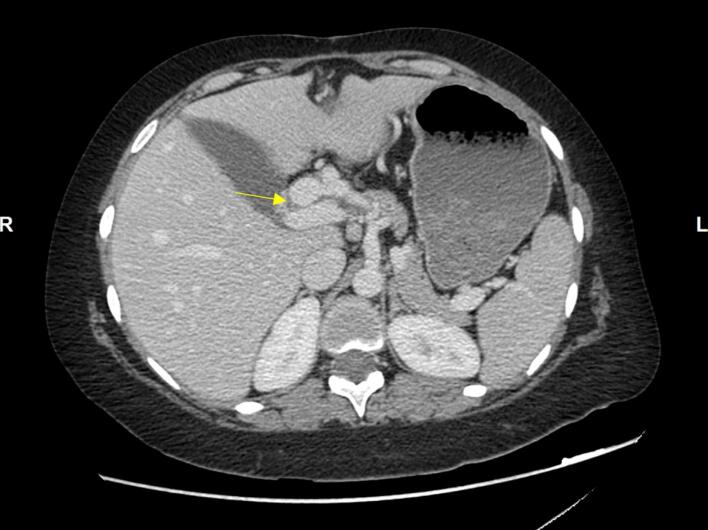
Arrow: hepatic artery aneurysm.

A follow-up CT scan of the abdomen in December 2023 revealed a saccular aneurysm measuring 18 × 17 mm originating from the proximal proper hepatic artery (Fig. 1).

**Fig. 2:**
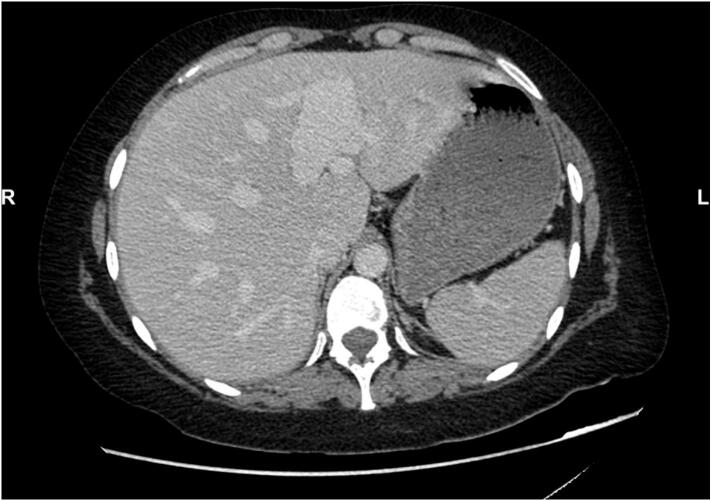
Portosystemic shunting secondary to arteriovenous fistula.

Additionally, a branch of the left hepatic artery was dilated (11 mm) and communicated with a significantly dilated distal left portal vein, associated with early arterial hyper-enhancement of the left hepatic lobe, suggestive of portosystemic shunting secondary to arteriovenous fistula (Fig. 2). Further investigations at that time (chest CT, brain MRI, labs) showed no abnormalities. No other sites of aneurysms were detected.

**Fig. 3:**
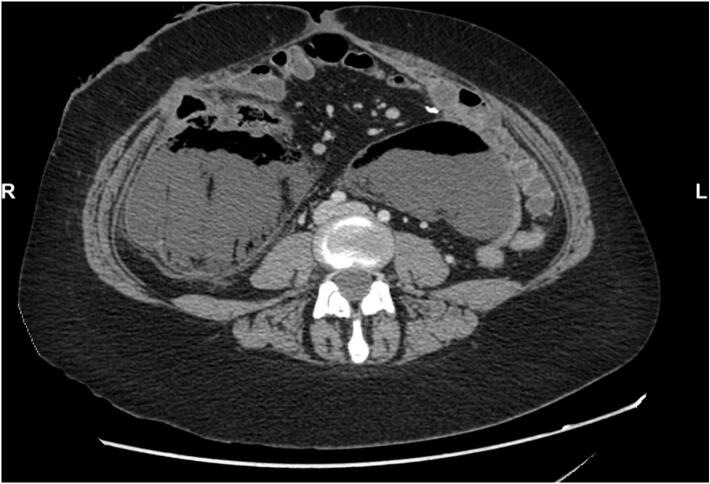
Abdominal collection due to bowel perforation.

In March 2024, the patient was readmitted for acute abdominal pain. A CT scan revealed a large abdominal collection, most likely due to a new bowel perforation (Fig. 3). The decision was made to perform bilateral drainage of the abdomen, and the patient was administered broad-spectrum antibiotics and intensive hydration.

On Day 4, despite optimal medical management, the patient presented severe abdominal pain, tachycardia, and hypotension, necessitating urgent transfer to the operating room for laparotomy.

During laparotomy, multiple transverse colon perforations with stercoral peritonitis and a retracted stoma were identified. Additionally, many small bowel perforations were found after an exhaustive adhesiolysis of the friable tissues. Multiple small bowel anastomoses were performed, and an extended colectomy was performed, alongside an ascending colostomy. Intensive abdominal lavage with more than 10 l of fluid was conducted, and three abdominal drains were placed.

The patient was then transferred to the ICU, where she received multiple transfusions due to significant blood loss during surgery. She underwent a course of several antibiotics for severe multiresistant multi-bacterial infection. Her ICU stay lasted for 7 days. On Day 5, she developed an enterocutaneous fistula with wound infection, which was managed conservatively with total parenteral nutrition, NG tube insertion, and wound care.

**Fig. 4:**
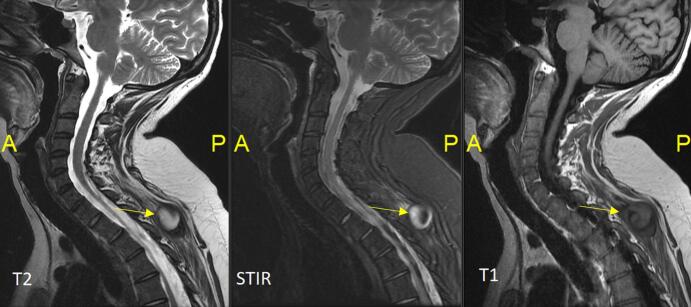
Arrow: Paraspinal aneurysm.

**Fig. 5:**
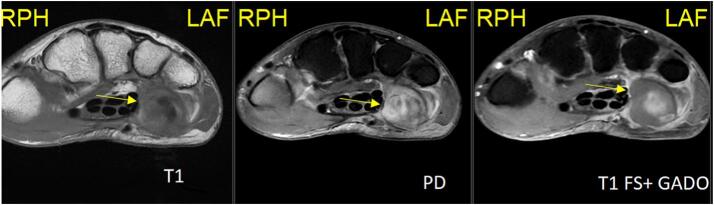
Arrow: Left wrist aneurysm.

**Fig. 6:**
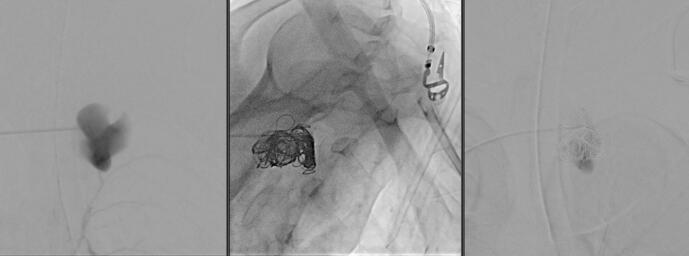
Coiling of aneurysm.

During her hospital stay, on Day 19, the patient experienced intense left arm and hand pain, particularly in the last two digits. Imaging revealed an aneurysm measuring 4.2 cm in the right paraspinal muscles between the spinous processes of T3 and T4 (Fig. 4), along with a 2 × 1 cm aneurysm at the level of the left wrist in the hypothenar compartment just after Guyon's canal (Fig. 5). This aneurysm, measuring approximately 20 × 10 mm, exerted a mass effect on the ulnar nerve at this level. A cervical MRI was also performed for posterior neck pain and showed a 25 × 21 mm false aneurysm in the right paraspinal muscles between the spinous processes of T3 and T4. A decision was made to intervene radiologically and embolize this aneurysm due to its risk of bleeding, using the coiling technique (Fig. 6). The wrist aneurysm was managed medically with physiotherapy and pain management.

**Fig. 7:**
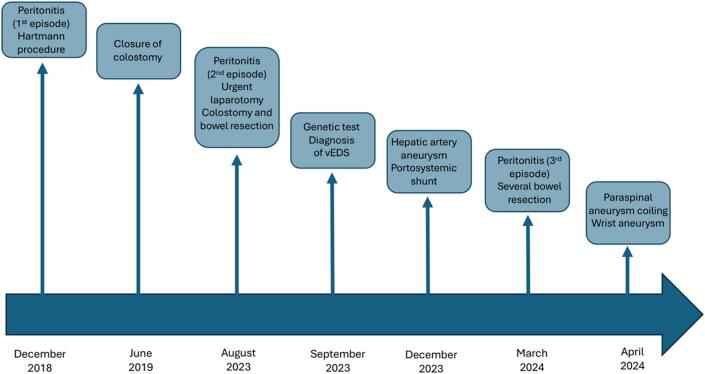
Timeline of clinical events and procedures.

The patient was then discharged six weeks later on pain medication, oral antibiotics, nutritional support, and wound management. She is being followed up by regular visits, labs, and imaging to prevent further complications. A timeline detailing the clinical events and procedures is presented in Fig. 7.

## 4 Genetic results

**Fig. 8:**
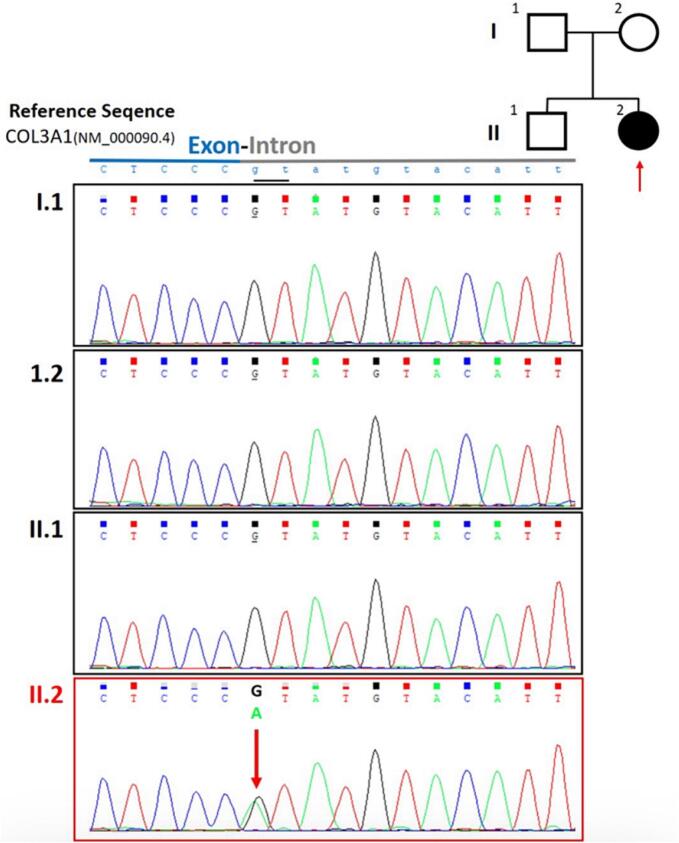
Pedigree of the family studied, the proband (II-2) is indicated by a shaded circle. Sanger sequencing confirmation for the identified COL3A1 variant by Whole Exome Sequencing (WES): Sequencing electropherograms of genomic DNA for the patient (II.2) showing the de novo heterozygous variant in the COL3A1 gene (NM_000090.3): c.1662 + 1G > A, and the other family members (I.1, I.2 and II.1) showing normal results.

At first, a whole exome sequencing (WES) analysis was requested for the patient (pedigree in Fig. 8, II.2) to screen for variants responsible for Ehlers-Danlos syndrome or other bleeding disorders, in a non-consanguineous family with no previous history of bleeding.

The results of WES sequencing showed a heterozygous splicing variation in the COL3A1 gene (NM_000090.3): c.1662 + 1G > A. This variant is predicted to disrupt the highly conserved donor splice site. It is classified as a class 1 Pathogenic variant according to the recommendations of the American College of Medical Genetics and Genomics (ACMG) [3]. Also, this variant is listed as pathogenic in Clinvar (Variation ID: 101269). WES result confirmed the genetic diagnosis of autosomal dominant vascular-type Ehlers-Danlos syndrome.

To offer genetic counseling for the family, it was important to establish the origin of the variant, inherited or de novo. After signing an informed consent form, targeted parental testing was performed on all family members. The Sanger sequencing method was used to validate the results in the patient (Fig. 8, II.1) and the patient's family members (Fig. 8, I.1, I.2 and II.1). Oligonucleotide primers were designed using the Primer 3.0 tool to amplify the exon23-intron junction of the COL3A1 gene. PCR amplification was carried out using the QIAGEN Multiplex PCR Master Mix. DNA sequencing was sequenced in both directions (Forward and Reverse). PCR products were purified and sequenced on the ABI 3500 Genetic Analyzer. The sequencing data were analyzed using the ChomasPro software, version 2.1.9.

The heterozygous COL3A1 variant was confirmed in the patient (Fig. 8, II.2), while none of the other family members carried the variant. This indicates that the variant is de novo, and was not inherited from either parent. This information was communicated to the family, explaining that it is a de novo variant, neither parent is a carrier, and there is no risk for the brother.

## 5 Discussion

Numerous cases of vascular Ehlers-Danlos Syndrome (vEDS) have been documented, often involving a history of recurrent bowel perforations [[4], [5], [6]], ruptured aneurysms [7], and various types of fistulas [8]. Our case stands out as a stark illustration of the severity inherent in this syndrome and the immense challenges it presents for management. The current case can help establish management plans in front of such presentations. This patient exhibited multiple rapidly growing aneurysms, some of which manifested symptoms due to their impact on nearby structures. Moreover, the last laparotomy underscored the daunting nature of this condition, with abundant necrotic tissue and exceedingly fragile materiel found in the bowel, mesentery, and skin. Such complexities emphasize the critical need for heightened vigilance and comprehensive care strategies in managing vEDS cases.

Our case was particularly noteworthy for several reasons: firstly, it involved a young patient with vascular Ehlers-Danlos disease, which differs from many case reports that typically describe older cases. Secondly, unlike many patients who are diagnosed post-mortem during autopsies following ruptured aneurysms and sudden death, our case was diagnosed during active management. Thirdly, the simultaneous presentation of bowel perforation and multiple aneurysms added to the complexity and uniqueness of this case. Lastly, the management course and the preventive measures taken have added value to the literature when encountering such different presentations.

The rapid diagnosis and vigilant monitoring of patients with vascular Ehlers-Danlos Syndrome (vEDS) are paramount, needing multiple emergency room visits for any new symptoms or pain in this population. Prompt management is crucial, underscoring the utmost importance placed on patient care and safety. This often entails a series of interventions, prolonged hospital stays, long-term antibiotic therapy, and meticulous attention to supporting proper nutrition, along with comprehensive patient counseling.

The medical management of vascular Ehlers-Danlos syndrome (vEDS) remains a subject of debate and lacks robust evidence. While medications such as celiprolol, a beta-blocker, have shown promise in reducing arterial events, the overall efficacy and safety of various medical therapies are not conclusively established. The limited number of studies and their small sample sizes make it challenging to develop definitive guidelines. Therefore, while some treatments show potential, their use should be considered cautiously and on an individual basis [9].

Vascular lesions of vEDS have traditionally been treated conservatively because of elevated rates of intraoperative and postoperative complications in this population [10]. While still not well established and lacking clear guidelines for management, elective surgical treatment of vascular disorders in EDS patients using open and endovascular procedures has been associated with good outcomes. Results suggest that vascular interventions in these EDS patients can be safely performed and should not be withheld until rupture or acute symptoms arise [11].

A multidisciplinary approach remains essential for the optimal management of vEDS, as it encompasses various medical specialties including digestive surgery, internal medicine, interventional radiology, and vascular surgery. Timely interventions are imperative, and any new symptoms experienced by the patient should be thoroughly investigated to prevent potential complications.

While vEDS is a rare condition, its significance cannot be understated, particularly in cases of recurrent intestinal perforations [12]. This case serves as a poignant reminder of the critical importance of early and exact diagnosis, genetic counseling, and the implementation of appropriate procedures in patients with vEDS [2].

Indeed, it is crucial to acknowledge the substantial burden and profound psychological impact on both the patient's mental health and their family dynamics. The constant fear of potential complications and the uncertainty surrounding the course of the disease can significantly exacerbate anxiety levels. This heightened state of anxiety not only affects the patient's overall well-being but also has ripple effects on their familial support system.

The persistent threat of life-threatening events such as bowel perforations or ruptured aneurysms looms large, casting a shadow over every aspect of daily life.

Moreover, the psychological impact extends beyond the individual patient, affecting the entire family unit. Addressing the psychological well-being of patients with vEDS and their families is therefore an integral part of comprehensive care. Supportive interventions, such as counseling, psychoeducation, and access to mental health resources, play a crucial role in mitigating the emotional strain associated with living with vEDS. By acknowledging and addressing these psychological challenges, healthcare providers can strive to enhance the overall quality of life for individuals and families affected by this complex condition.

## 6 Conclusion

In conclusion, this case exemplifies a distinctive presentation and management of vascular Ehlers-Danlos syndrome (vEDS). The primary aim is to heighten awareness, encourage early diagnosis, and disseminate management strategies. A collaborative, multidisciplinary approach is crucial in effectively handling such complex cases while emphasizing the importance of active patient and caregiver involvement in the management plan.

## Informed consent statement

Written informed consent was obtained from the patient and all family members for conducting the genetic study and for the publication of this case report, including accompanying data and images.

## Funding

No funding was needed in this study.

## Ethical approval

Patient provided consent to share the medical records.

Ethical approval for this study (Ethical Committee N3671) was provided by the Ethical Committee of Hotel Dieu de France University Hospital on the 6th of April 2024.

## CRediT authorship contribution statement

Farid Abi Habib: Data analysis and writing the paper.

Michael Osseis: Study concept and design, and writing the paper.

Alain Chebly: Genetic analysis and interpretation.

Elie El Feghali: Reviewing the paper.

Roger Noun: Reviewing the paper.

## Guarantor

Farid Abi Habib.

## Declaration of competing interest

The authors have nothing to disclose.
